# 

**Published:** 1951-10

**Authors:** 


### Local Medical Notes

HONOUR

*O.B.E.*—Dr. Elizabeth Casson.

APPOINTMENTS

Jane Philp, M.B.—Assistant Anaesthetist, Warrington.

G. H. Wattley, M.D., M.R.C.P., D.T.M. & H.—M.O., Grade A, Trinidad.

BRISTOL : N. J. Brown, M.R.C.P.—Consultant Pathologist.
T. J. Butler, F.R.C.S.—Consultant Surgeon.
K. H. Gaskell, D.M.R.D.—Consultant Radiologist
D. M. Milne, F.R.C.S.—Consultant Thoracic Surgeon.
E. C. Turton, M.B., M.Sc., D.P.M.—Consultant Physician in Psychiatry.
Pamela Westhead, M.B., B.Ch., D.A., Consultant Anaesthetist.

B. A. Jenkins, M.D., M.R.C.P., Consultant Physician, West Cornwall.

H. R. Jolly, M.D., M.R.C.P., D.C.H., Consultant Paediatrician, Plymouth.

A. E. Jowett, O.B.E., M.B., F.R.C.S., Consultant Orthopaedic Surgeon, South Somerset.

J. E. Tees, M.R.C.S., D.A.—Anaesthetist, North Gloucestershire.

R. B. H. Tierney, M.B., Consultant Pathologist, Exeter.

H. R. E. Wallis, M.R.C.P., D.C.H.—Consultant Paediatrician, Bath.

A. E. Wilson, D.O.M.S.—Consultant Ophthalmologist, South Somerset.

UNIVERSITY OF BRISTOL

PRIZES

*Edgeworth.*—Miss M. E. Moore.

*Mackie.*—W. G. Benson.

*Western Dental Company's.*—J. E. Bowerman.

*Phillips.*—J. P. Fletcher.

*Crosby Leonard.*—Miss M. E. Barton.

*Tibbitts.*—P. Maton Terry.

### Degrees and Diplomas

**University of Bristol.**

M.D.—M. M. Lewis.

M.B., Ch.B. (*with Second Class Honours*).—D. R. Coles (with Distinction in Public Health and in Child Health), June K. Lloyd (with Distinction in Public Health and in Surgery).

M.B., Ch.B.—Margaret M. Ashton, W. G. Benson (with Distinction in Public Health), A. C. Brown, C. T. Brown, Ruth E. Coles, T. E. Dada, C. E. Dickson, Dorothy R. Douglas, M. S. Dunnill (with Distinction in Surgery), M. A Feldman, D. H. Cox, B. W. Hill, Doreen E. D. Hillier, J. A. Jefferies, S. J. Lloyd, Ruth M. Martin, S. J. Palmer, M. E. Parry, D. S. Paton, Margaret R. Salisbury, J. H. Scudamore, M. N. Simmonds, R. E. D. Simpson, R. A. Stride, A. M. Sweeting, Denorah Vardy, P. G. J. Wilcock.

D.P.M.—O. Lyth, W. D. C. Thomas.

L.D.S.—M. F. Flint, Ruth Mulhern.

M.B., Ch.B.—*Second Examination.*—Pamela M. Atkinson, M. T. Charleston, J. F. Daintree, D. J. C. Felton, D. B. Fowler, J. K. Howell, J. S. Hughes-Games, Jean D. Matthews, J. M. V. Packer, Ruth D. Penn, P. J. Roylance, R. M. Stone, D. H. Waldron, G. C. B. Winter.

**Royal College of Surgeons.**—F.R.C.S.: P. A. James, M.B., Ch.B.

**Royal Colleges of Physicians and Surgeons (Conjoint Board).**—M.R.C.S., L.R.C.P.: Margaret M. Ashton, T. E. Dada, D. H. Fox, B. W. Hill.

### 1951 Index to Vol. LXVIII

Alexander, G. L.—Surgical Neurology, 55

Andrews, C. T.—Geriatrics, 35

Animal Disease in Relation to Man.—A. M. G. Campbell, 105

Apley, J.—Congenital Heart Disease, 124

Appointments, 33, 62, 102, 144

Atomic Bomb, The.—R. C. Tudway and J. H. Challenger, 71

Backache, Sciatica and Ruptured Discs.—K. Pridie, 95

Balance Sheets, etc., 28, 140

Blood Transfusion (Editorial), 25

Bomb, *see* Atomic

Campbell, A. M. G.—Animal Diseases, 105

Challenger, J. H.—Atomic Bomb, 79

Congenital, *see* Heart

Corner, B.—Streptomycin, 16

Davy, Sir Humphry—Memorial to, 32

Degrees and Diplomas, 34, 63, 103, 145

Discs, Ruptured, *see* Backache

Editorial: The Presidency; Blood Transfusion, 25; Our Function; Old Age, 98; New Editor, 139

Editor's Report, 27, 139

Food Infection Problems, Some: Long Fox Lecture.—Sir William Savage, 9

Fox.—XXXIX Long Fox Memorial Lecture, 9; XL Lecture, 102

Geriatrics.—C. T. Andrews, T. S. Wilson and W. Hughes, 35
(*See also* Editorial)

Heart Disease, Congenital.—R. Milnes Walker and J. Apley, 124

Hughes, W.—Geriatrics, 42

In Memoriam, 26, 58

Jackman, W. A.—Some Surgical Milestones (P.A.), 1

*Journal*, The.—E. Watson-Williams, 116; *and see* Editorial, etc.

Keratosis Pharyngis. — E. Watson-Williams, 23

Liver Function Tests.—G. Kemp McGowan, 46

Local Medical Notes, 32, 62, 102, 144

Long Fox Lecture, *see* Fox

Mason, S.—Streptomycin, 16

McGowan, G. K.—Liver Function Tests, 46

McLachlan, A. E. W.—Reiter's Syndrome, 87

Maternity Hospital, Opening, 32

Neurology, Surgical.—G. L. Alexander, 55

Nixon, J. A.—Obituary, 58

Obituary: J. A. Nixon, 58

Old Age: Editorial, 99

President's Address: Some Surgical Milestones, 1

Presidency, The: Editorial, 25

Pridie, K.—Backache, Sciatica and Ruptured Discs, 95

Prizes, 34, 103, 144

Reiter's Syndrome.—A. E. W. McLachlan, 87

Savage, Sir William.—Some Food Infection Problems, 9

Sciatica, *see* Backache

Societies, Meetings of:

Bristol Med.-Chir. Soc., 27, 60, 100, 139

B.M.A.:

Bath, Bristol and Somerset Branch, 31, 62, 101

Gloucestershire Branch, 31

Bristol Division, 62, 102

Newport Division, 143

West Somerset Division, 62

Devon and Exeter Med.-Chir. Soc., 30, 61

Plymouth Med. Soc., 30

South-Western Laryngological Assn., 32, 61, 101

S. Wales Soc. of Anaesthetists, 143

Welsh Paediatric Club, 143

West of England and Wales Soc. Dermatology, 31

West Somerset Medical Club, 143

Streptomycin.—B. Corner and S. Mason, 16

Surgical Milestones, Some (P.A.).—W. A. Jackman, 1

Surgical Neurology.—G. L. Alexander, 55

Subscribers, List of, 64

Thoracic Surgery.—Belsey, 101; *and see* Heart Disease

Transfusion.—Editorial, 25

Tudway, R. C.—Atomic Bomb, 71

Walker, R. Milnes.—Congenital Heart Disease, 133

Watson-Williams, E.—The *Journal*, 116; Keratosis Pharyngis, 23

Wilson, T. S.—Geriatrics, 38

